# A comparison study between gross tumor volumes defined by preoperative magnetic resonance imaging, postoperative specimens, and tumor bed for radiotherapy after breast-conserving surgery

**DOI:** 10.1097/MD.0000000000005839

**Published:** 2017-01-13

**Authors:** Aiping Zhang, Jianbin Li, Wei Wang, Yongsheng Wang, Dianbin Mu, Zhaoqiu Chen, Qian Shao, Fengxiang Li

**Affiliations:** a Medicine and Life Sciences College of Shandong Academy of Medical Sciences, Jinan University; b Department of Radiation Oncology; c Breast Cancer Center; d Department of Pathology; e Department of Radiology, Shandong Cancer Hospital Affiliated to Shandong University, Jinan, Shandong Province, China.

**Keywords:** breast-conserving surgery, excised specimen, preoperative magnetic resonance imaging, radiotherapy, tumor bed

## Abstract

**Background:**

The identification and contouring of target volume is important for breast-conserving therapy. The aim of the study was to compare preoperative magnetic resonance imaging (MRI), postoperative pathology, excised specimens’ (ES) size, and tumor bed (TB) delineation as methods for determining the gross tumor volume (GTV) for radiotherapy after breast-conserving surgery (BCS).

**Methods:**

Thirty-three patients with breast cancer who underwent preoperative MRI and radiotherapy after BCS were enrolled. The GTVs determined by MRI, pathology, and the ES were defined as GTV_MRI_, GTV_PAT_, and GTV_ES_, respectively. GTV_MRI+1_ was defined as a 1.0-cm margin around the GTV_MRI_. The radiation oncologist delineated GTV of the TB (GTV_TB_) using planning computed tomography according to ≥5 surgical clips placed in the lumpectomy cavity (LC).

**Results:**

The median GTV_MRI_, GTV_MRI+1_, GTV_PAT_, GTV_ES_, and GTV_TB_ were 0.97 cm^3^ (range, 0.01–6.88), 12.58 cm^3^ (range, 3.90–34.13), 0.97 cm^3^ (range, 0.01–6.36), 15.46 cm^3^ (range, 1.15–70.69), and 19.24 cm^3^ (range, 4.72–54.33), respectively. There were no significant differences between GTV_MRI_ and GTV_PAT_, GTV_MRI+1_ and GTV_ES_, GTV_ES_ and GTV_TB_ (*P* = 0.188, 0.070, and 0.264, respectively). GTV_MRI_ is positively related with GTV_PAT_. However, neither GTV_ES_ nor GTV_TB_ correlated with GTV_MRI_ (*P* = 0.071 and 0.378, respectively). Furthermore, neither GTV_ES_ nor GTV_TB_ correlated with GTV_MRI+1_ (*P* = 0.068 and 0.375, respectively).

**Conclusion:**

When ≥5 surgical clips were placed in the LC for BCS, the volume of TB was consistent with the volume of ES. Neither the volume of TB nor the volume of ES correlated significantly with the volume of tumor defined by preoperative MRI.

## 1 Introduction

Breast-conserving therapy is the standard treatment in patients with early-stage breast cancer.^[1]^ A meta-analysis conducted by the Early Breast Cancer Trialists’ Collaborative Group revealed that radiotherapy after breast-conserving surgery (BCS) halved the local recurrence rate and reduced the mortality rate.^[2]^ During breast radiotherapy, boost irradiation to the tumor bed (TB) can reduce ipsilateral breast tumor recurrences, especially in young patients or those with a high risk of recurrence.^[3,4]^ Therefore, the identification and contouring of the TB based on surgical clips and/or the seroma is important for boost irradiation.

Because most ipsilateral breast cancer recurrences occur in or nearby the TB, accelerated partial breast irradiation (APBI) has gained popularity in patients with low local recurrence risk.^[5,6]^ External-beam partial breast irradiation (EB-PBI) is one such approach, and the clinical impact of accurate TB delineation according to the boundary of lumpectomy cavity (LC) is paramount when using EB-PBI.^[7,8]^ For a selected group of early-stage breast cancer patients, Polgár et al^[9]^ found ipsilateral breast tumor recurrence rates among patients treated by EB-PBI to be similar to those treated with whole breast irradiation. However, a recent study demonstrated that EB-PBI increased the rates of adverse cosmesis and late-radiation toxicity compared with standard whole breast irradiation.^[10]^ Therefore, the procedures for defining and delineating TB volume should be reviewed.

Compared to conventional imaging modalities such as mammography and ultrasonography, magnetic resonance imaging (MRI) has superior sensitivity and accuracy for the detection and visualization of tumor extent.^[11–14]^ Furthermore, because of high spatial resolution, preoperative MRI can detect occult tumors and provide additional information about the original tumor location.^[15–17]^ However, whether surgeons and radiation oncologists perform surgery or determine irradiation target volume based on preoperative MRI-derived parameters has not been widely determined. In addition, the relationships among preoperative imaging and surgical management, preoperative imaging, and TB delineation have been reported.^[16,18]^ However, a comparison of tumor volumes derived from preoperative imaging, postoperative specimen analysis, and TB delineation has not been investigated. The aim of this study was to explore gross tumor volume (GTV) differences and correlations according to preoperative breast MRI, postoperative specimen analysis, and TB delineated using surgical clips for radiotherapy after BCS.

## 2 Materials and methods

### 2.1 Patients and selection

The female patients with pathology-proven breast cancer diagnosed between April 2014 and March 2015 and who were eligible for BCS were recruited. Enrolled patients underwent preoperative MRI and had clinical T1-2N0M0 stage cancers. Eligible patients included those who underwent lumpectomy and had tumor negative margins during a single operation. To improve the delineation accuracy and consistency, all of the enrolled patients had seroma clarity score of 3 to 5 and ≥5 surgical clips fixed to the central bottom and lateral edges of the excision cavity to mark the LC boundaries. Patients with a history of ipsilateral breast surgery and chest radiotherapy were excluded from the recruitment, patients with oncoplastic BCS were excluded from analysis, and patients who received neoadjuvant chemotherapy or neoadjuvant endocrine therapy were also excluded. This study was approved by the Institutional Review Board (Shandong Tumor Hospital Ethics Committee). Written informed consent was obtained from all patients.

### 2.2 Magnetic resonance imaging

MRI was performed using the Philips Achieva 3.0-T scanner (Amsterdam, Netherlands) with the THRIVE (T1 high-resolution isotropic volume excitation) acquisition technique. Patients were placed prone with the breasts positioned in a dedicated bilateral breast coil. The diagnostic MRI protocol began with preliminary imaging using fast-spin echo sagittal T2 with fat saturation and axial T1 sequences. This was followed by dynamic high-resolution simultaneous imaging of both breasts using the THRIVE sequence with 8 dynamic scans with fat saturation, performed after intravenous administration of a contrast agent (gadopentetate dimeglumine, 0.1 mmol/kg). Postprocessing consisted of 2 series of subtraction images. The subtraction images that were transferred to MIMvista version 6.1.0 (MIM Software; Cleveland, OH) software were 3 mm thick.

### 2.3 Specimen processing

The pathologists who evaluated the surgical specimens were blinded to the positions of the MRI-planned excision margins. Unfixed excised specimens (ES) were placed in a graduated cylinder, and GTV (GTV_ES_) was determined using the Archimedes principle. The maximum length (cm), width (cm), and height (cm) of the tumor were measured by an experienced pathologist. The volume of tumor (GTV_PAT_) was calculated using the following equation: GTV_PAT_ = 1/6π × length × width × height.

### 2.4 Acquisition of computed tomography image sets

Before radiotherapy, all patients underwent a planning computed tomography (CT) scan in the supine position with the arms extended above the head. The standard CT simulation was acquired with a thickness of 3 mm using a 16-slice Brilliance Big Bore CT scanner (Philips Medical Systems, Inc.; Cleveland, OH). Subsequently, planning CT image sets were transferred to the Eclipse treatment planning system (Eclipse 8.6, Varian Medical Systems; Palo Alto, CA) for structure delineation.

### 2.5 Target volume delineation

Tumor volume (cm^3^) according to MRI (GTV_MRI_) was delineated by the same experienced radiologist using MIMvista software (MIM 6.1.0) with information from the preoperative MRI subtraction images. We also reconstructed the volume by adding a 1.0-cm margin around the GTV_MRI_ (GTV_MRI+1_) to match the 1.0-cm margin routinely applied during surgery in our hospital.

To eliminate interobserver variation, the GTV_TB_ was contoured by the same breast irradiation oncologist specializing in radiation treatment of breast carcinoma with more than 5 years of radiotherapy experience in the Eclipse treatment planning system using the placement of the surgical clips as a guideline.

### 2.6 Statistical analyses

Because of non-normal distribution of variables, median values and ranges were used to describe the data. A Wilcoxon signed-rank test was performed to compare the paired tumor volume variables. Correlations were studied using the Spearman rank correlation coefficient. Statistical analyses were conducted using SPSS Statistics version 17.0 (Chicago, Illinois, America). All statistical tests were 2-sided, and *P* value <0.05 was considered statistically significant.

## 3 Results

### 3.1 Patient characteristics

There were 40 patients enrolled in our study. Seven patients were excluded from analysis because 3 patients were with oncoplasty and 4 patients received neoadjuvant chemotherapy or neoadjuvant endocrine therapy. The remaining 33 patients were eligible for further analysis. Patient and tumor characteristics are shown in Table 1. Most patients were diagnosed with an invasive ductal carcinoma with or without ductal carcinoma in situ (94%). Pathological stage was predominantly T1 (64%). All patients underwent lumpectomy. The average interval from lumpectomy to the planning CT scan was 91 days (range, 19–172 days).

**Table 1:**
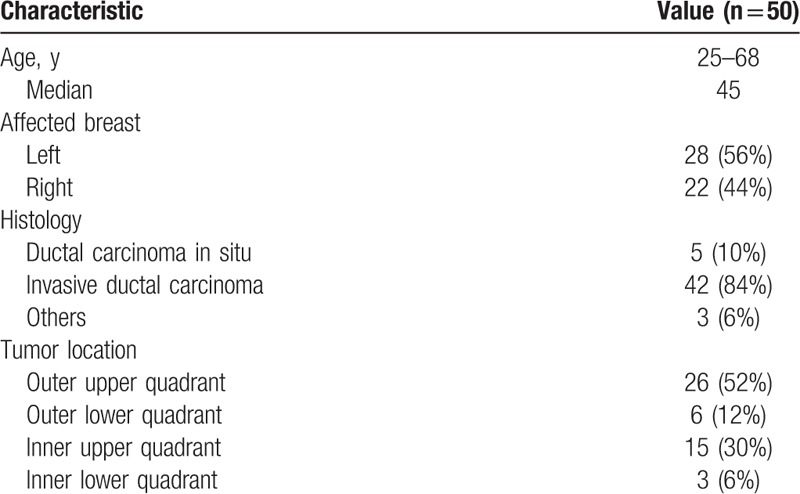
Patient characteristics.

### 3.2 Comparisons of gross tumor volumes

**Figure 1:**
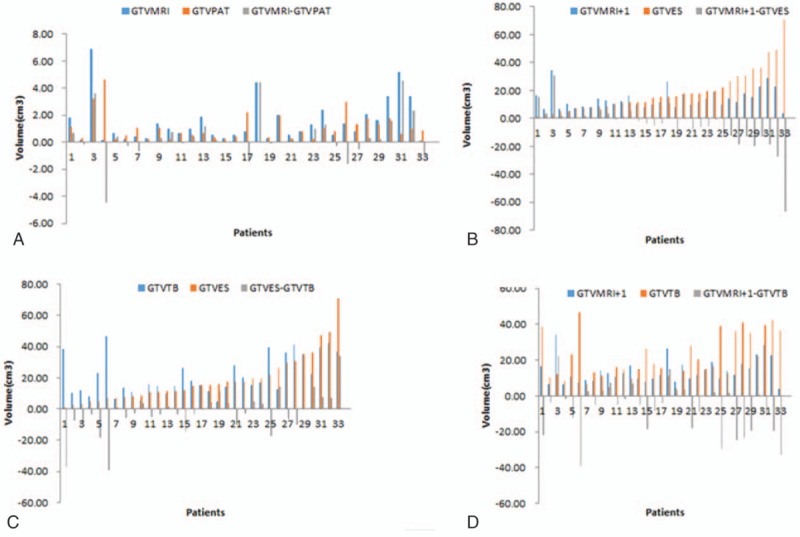
Distribution of gross tumor volume (GTV) and volume difference between (A) preoperative magnetic resonance imaging (GTV_MRI_) and pathology (GTV_PAT_), (B) extending 1.0-cm margin around the GTV_MRI_ (GTV_MRI+1_) and excised specimens (GTV_ES_), (C) excised specimens (GTV_ES_) and tumor bed (GTV_TB_), (D) extending 1.0-cm margin around the GTV_MRI_ (GTV_MRI+1_) and tumor bed (GTV_TB_).

The median GTVs are shown in Table 2. GTV_TB_ was significantly larger than GTV_MRI_ or GTV_PAT_ or GTV_MRI+1_ (*P* = 0.000, 0.000, and 0.007, respectively). There were no significant differences between GTV_MRI_ and GTV_PAT_, or between GTV_MRI+1_ and GTV_ES_ (*P* = 0.188 and 0.070, respectively). Furthermore, there was no significant difference between GTV_ES_ and GTV_TB_ (*P* = 0.264). Figure 1 shows the distribution of volume and volume difference between GTV_MRI_ and GTV_PAT_ (Fig. 1A), between GTV_MRI+1_ and GTV_ES_ (Fig. 1B), between GTV_ES_ and GTV_TB_ (Fig. 1C), and between GTV_MRI+1_ and GTV_TB_ (Fig. 1D).

**Table 2:**
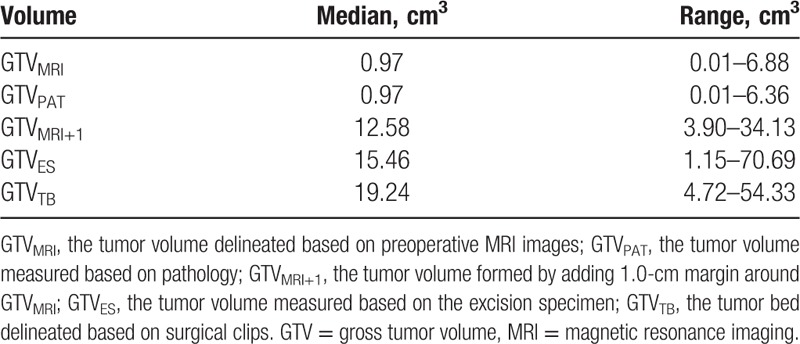
Gross tumor volumes.

### 3.3 Correlations of gross tumor volumes

**Figure 2:**
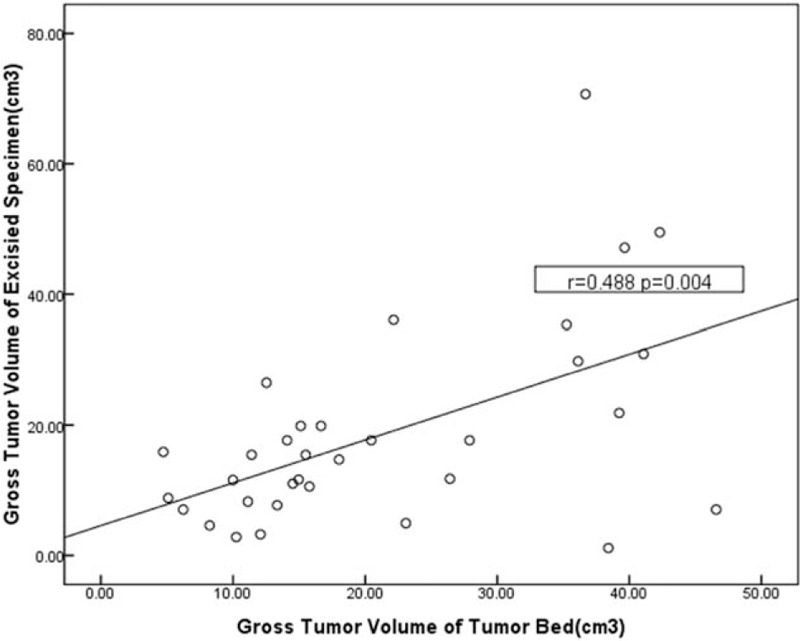
The relationship of gross tumor volume (GTV) between excised specimens (GTV_ES_) and tumor bed (GTV_TB_).

There was no significant correlation between GTV_MRI_ and GTV_ES_, or between GTV_MRI_ and GTV_TB_. Similarly, there was no significant correlation between GTV_MRI+1_ and GTV_ES_, or between GTV_MRI+1_ and GTV_TB_. Figure 2 shows the relationship between GTV_ES_ and GTV_TB_. GTV_ES_ was positively related with GTV_TB_.

The ratio of GTV_ES_ to GTV_TB_ represented the coincidence degree between GTV_ES_ and GTV_TB_, and its median was 0.83. The relation between the ratio of GTV_ES_ to GTV_TB_ and the interval duration, number of surgical clips utilized, and primary tumors’ locations were determined. The median number of surgical clips was 5 (range, 5–7). There were no significant correlations between the ratio of GTV_ES_ to GTV_TB_ and any of these factors (*P* > 0.050 for all).

## 4 Discussion

For BCS, it is essential to obtain negative margins, as margin status is an important prognostic factor for local recurrence after breast-conserving therapy.^[19]^ However, excising large masses of breast tissue could jeopardize cosmetic outcome and has not been shown to provide better local control or to improve overall survival rates.^[20]^ Preoperative imaging-guided techniques were effective in improving the definition of the extent and localization of the tumor.^[21]^ Furthermore, compared to postoperative EB-PBI, preoperative target volume delineation leads to considerably less interobserver variation.^[22,23]^ Hence, in order to improve the balance between local control and cosmesis outcome, it is necessary to ascertain the extent of the tumor as accurately as possible by preoperative imaging.

MRI is currently used to evaluate disease extent for BCS, and its role in the evaluation of breast lesions is evolving. MRI has been shown to detect multifocal and multicentric cancers more often than conventional imaging.^[11,24]^ Moreover, Bilimoria et al^[25]^ evaluated the effect of breast MRI on clinical management and reported that 9.7% of women had a beneficial modification in surgical management based on preoperative breast MRI. Moreover, the rationale for preoperative MRI was that accurate delineation of the tumor extent might allow surgeons to achieve a negative resection margin during a single operation. Therefore, we performed a comparative study of volume relationships to evaluate the influence of preoperative MRI-based determination of tumor extent and target volume delineation on radiotherapy.

Several studies, utilizing various methods, have evaluated the accuracy of MRI for assessing tumor size and have shown a range of correlations between MRI and pathology.^[26,27]^ In 100 (53%) patients with breast cancer tumors, Grimsby et al^[27]^ reported that GTV_PAT_ and GTV_MRI_ were concordant within 0.5 cm. Similarly, there was no significant difference between GTV_MRI_ and GTV_PAT_ in the present study. Because of a lack of pathologic validation, diagnosing breast cancer lesions by MRI alone could produce false-positive or false-negative results.^[28,29]^ In the present study, pathological analysis was performed to avoid false-positive or false-negative results, thereby validating the accuracy of MRI.

Although a few studies have investigated the accuracy of MRI to depict disease extent,^[12,13]^ it is unclear how often surgeons perform surgery based on preoperative MRI-obtained parameters. In the present study, GTV_MRI_ was not significantly correlated with GTV_ES_. Considering the fact that lumpectomy was performed with a circumferential margin of at least 1.0 cm,^[30]^ we analyzed the relationship between GTV_MRI+1_ and GTV_ES_. While there was no statistically significant difference between GTV_MRI+1_ and GTV_ES_, they were not correlated with each other either. Because of non-normal distribution of variables, the standard deviation (SD) of GTV_ES_ (SD = 15.30) was larger than GTV_MRI+1_ (SD = 6.89). This result indicated that the distribution of GTV_ES_ was more discrete. As the accuracy of MRI was confirmed by pathological analysis, this seemingly contradictory finding can be explained by the fact that surgeons perform surgical excision according to their experience, and intersurgeon variability also plays a significant role. Moreover, because surgeons performed BCS randomly and radiation oncologists delineated GTV_TB_ according to the surgical clips placed in the LC, neither GTV_MRI_ nor GTV_MRI+1_ correlated with GTV_TB_. Our results confirmed that the majority of surgeons performed BCS subjectively, ignoring imaging-guided diagnosis of tumor extent. Furthermore, in ES, the boundary of the primary tumor and surgical margin was anisotropic. Therefore, both surgeons and radiation oncologists should value the usefulness of preoperative MRI-guided techniques for surgical excision and GTV delineation.

Achieving accurate delineation of GTV_TB_ based on the LC size is critical for adequate local control in APBI. However, owing to breast density, the ability to visualize the LC is poor, and surgical clips and/or the seroma are therefore used to provide additional information on location.^[31]^ Dzhugashvili et al^[32]^ reported that the placement of surgical clips at lumpectomy enables visualization of the LC and improves the cavity visualization score on planning CT scans for APBI. However, there were no standard recommendations for the optimal number of markers to be implanted in the LC. Kirby et al^[22]^ reported that 5 or more implanted markers are likely to be adequate for the purposes of TB delineation for partial breast/breast boost radiotherapy. Based on this, TB volumes were outlined using ≥5 clips in our study. When we compared the GTV_TB_, delineated by surgical clips, to the GTV_ES_, there was no significant difference, and GTV_TB_ correlated significantly with GTV_ES_ (Fig. 2). This indicates that placement of ≥5 surgical clips at the cardinal points of the LC is extremely useful for the LC visualization and accurate for TB delineation.

Previous studies have reported that an increased duration between surgery and radiotherapy caused a decrease in seroma clarity and LC volume; this affected the GTV_TB_.^[33,34]^ However, whether the use of surgical clips to delineate the GTV_TB_ has a similar effect was uncertain. Hurkmans et al^[35]^ found that clip position could still change significantly after surgery, particularly when the initial seroma volume is large. Conversely, in the present study, the ratio of GTV_ES_ to GTV_TB_ was stable and was not affected by the length of duration between surgery and radiotherapy or the number of surgical clips used (for ≥5 clips). Furthermore, as a nonrigid tissue, the breast is deformed by gravity and breathing; therefore, the 3-dimensional movement correlations were asymmetrical. Hence, the location of the tumor according to breast quadrant might also influence the ratio of GTV_ES_ to GTV_TB_. However, tumor location was not associated with the ratio of GTV_ES_ to GTV_TB_ in this study. Overall, because surgeons and radiation oncologists did not value the role of preoperative imaging-guided diagnosis of tumor extent, it is likely that these factors had no effect on the ratio of GTV_ES_ to GTV_TB_.

If the accuracy of surgical resection could be improved by preoperative images, this is expected to further reduce the target volume for radiotherapy and result in a better cosmetic outcome alleviating postsurgical psychological stress. The potential limitation of this study is that it emphasized that most surgeons ignore imaging-guided diagnosis of tumor extent, but does not evaluate long-term outcomes in terms of local control and cosmesis outcome. Thus, in order to evaluate the usefulness of preoperative images in delineating tumor extent and TB, it is imperative that future studies should assess long-term results of ipsilateral breast tumor recurrences and cosmesis outcome.

## 5 Conclusion

Although preoperative MRI was available for every BCS patient, neither the volume of TB nor the volume of ES correlated significantly with the volume of tumor defined by the preoperative MRI. When ≥5 surgical clips were used to demarcate the LC during BCS, the volume of TB was consistent with the volume of ES. Therefore, a reasonably resected boundary of lumpectomy is a reliable indicator of the volume of TB. These data suggest that surgeons should strictly refer to preoperative images when performing surgical resections. Improving the accuracy of the volume of TB delineation can reduce the irradiated volume of normal breast tissue, achieving optimal oncologic and aesthetic outcomes.
